# Effects of Ambient Air Pollution on Precocious Puberty: A Case-Crossover Analysis in Nanjing, China

**DOI:** 10.3390/jcm12010282

**Published:** 2022-12-29

**Authors:** Haibo Yang, Aichen Ge, Hang Xie, Wei Li, Yizhou Qin, Wentao Yang, Dandan Wang, Wei Gu, Xu Wang

**Affiliations:** 1 Department of Emergency, Pediatric Intensive Care Unit, Children’s Hospital of Nanjing Medical University, Nanjing 210008, China; 2 Department of Science and Technology, Children’s Hospital of Nanjing Medical University, Nanjing 210008, China; 3 Department of Clinical Research, Children’s Hospital of Nanjing Medical University, Nanjing 210008, China; 4 Department of Quality Management, Children’s Hospital of Nanjing Medical University, Nanjing 210008, China; 5 School of Biomedical Engineering and Information, Nanjing Medical University, Nanjing 211166, China; 6 Department of Endocrinology, Children’s Hospital of Nanjing Medical University, Nanjing 210008, China

**Keywords:** air pollution, precocious puberty, distributed lag nonlinear model, case-crossover analysis, PM_2.5_, PM_10_

## Abstract

Background: Ambient air pollution is closely related to a variety of health outcomes. Few studies have focused on the correlations between air pollution exposure and children’s sexual development. In this study, we investigated the potential effects of exposure to air pollution on precocious puberty among children using real-world evidence. Methods: We conducted a case-crossover study (n = 2201) to investigate the effect of ambient air pollution exposure on precocious puberty from January 2016 to December 2021. Average exposure levels of PM_2.5_, PM_10_, SO_2_, NO_2_, CO, and O_3_ before diagnosis were calculated by using the inverse distance weighting (IDW) method. Distributed lag nonlinear model (DLNM) was used to assess the effect of air pollutants exposure on precocious puberty. Results: The mean age of the children who were diagnosed with precocious puberty was 7.47 ± 1.24 years. The average concentration of PM_2.5_ and PM_10_ were 38.81 ± 26.36 μg/m^3^ and 69.77 ± 41.07 μg/m^3^, respectively. We found that exposure to high concentrations of PM_2.5_ and PM_10_ might increase the risk of precocious puberty using the DLNM model adjusted for the age, SO_2_, NO_2_, CO, and O_3_ levels. The strongest effects of the PM_2.5_ and PM_10_ on precocious puberty were observed in lag 27 (OR = 1.72, 95% CI: 1.01–2.92) and lag 16 (OR = 1.95, 95% CI: 1.33–2.85), respectively. Conclusion: Our findings supported that short-term exposure to air pollution was the risk factor for precocious puberty. Every effort should be made to protect children from air pollution.

## 1. Introduction

Ambient air pollution is a mixture of particulate matter (PM) and gaseous pollutants from both human-made and natural sources, mainly including sulfur dioxide (SO_2_), nitrogen dioxide (NO_2_), carbon monoxide (CO), and ozone (O_3_). Ambient air pollution can enter human blood circulation through respiration, distribute to various organs, and lead to a variety of health outcomes [1,2]. In recent years, more and more studies have focused on the effects of air pollution exposure on children’s health [3,4,5]. However, few studies have focused on the correlations between air pollution exposure and children’s sexual development.

Particulate matter in the respirable range can carry a variety of polycyclic aromatic hydrocarbons and heavy metals, a group of compounds that include various endocrine disruptors which have a negative effect on reproductive health [6,7,8]. Sexual development is an important part of reproductive health. Precocious puberty is the most common sexual development disease among children which can lead to the early emergence of secondary sexual characteristics, early menarche, psychological behavior changes, and fusion of epiphyseal resulting in short adult stature. Childhood and adolescence are critical periods of sexual development when the endocrine system is extremely sensitive to environmental chemicals. Previous studies had shown that exposure to air pollution can lead to changes in endocrine hormone levels in children, but the biological effects of different pollutants may be different, which caused a combined effect [9,10].

Few studies have focused on the relationship between air pollution and children’s precocious puberty, as well as less attention has been paid to the critical window period when air pollution leads to children’s precocious puberty. In addition, although some studies had suggested that a combination of chemicals may affect sexual development and diseases [11], most of the population studies had focused on the effect of exposure to a single pollutant on sexual development and diseases, without considering the possibility of other gaseous pollutants as confounding factors [12,13,14].

In this study, we investigated the potential effects of children’s exposure to air pollution on precocious puberty in Nanjing, China. We focused on air pollution that was known or suspected to be harmful, including PM_2.5_ and PM_10_. Using real-world evidence, we assessed the associations of each individual air pollution exposure with precocious puberty in different exposure windows as well as considered SO_2_, NO_2_, CO, and O_3_ as confounding factors.

## 2. Methods

### 2.1. Study Design and Participants

**Figure 1:**
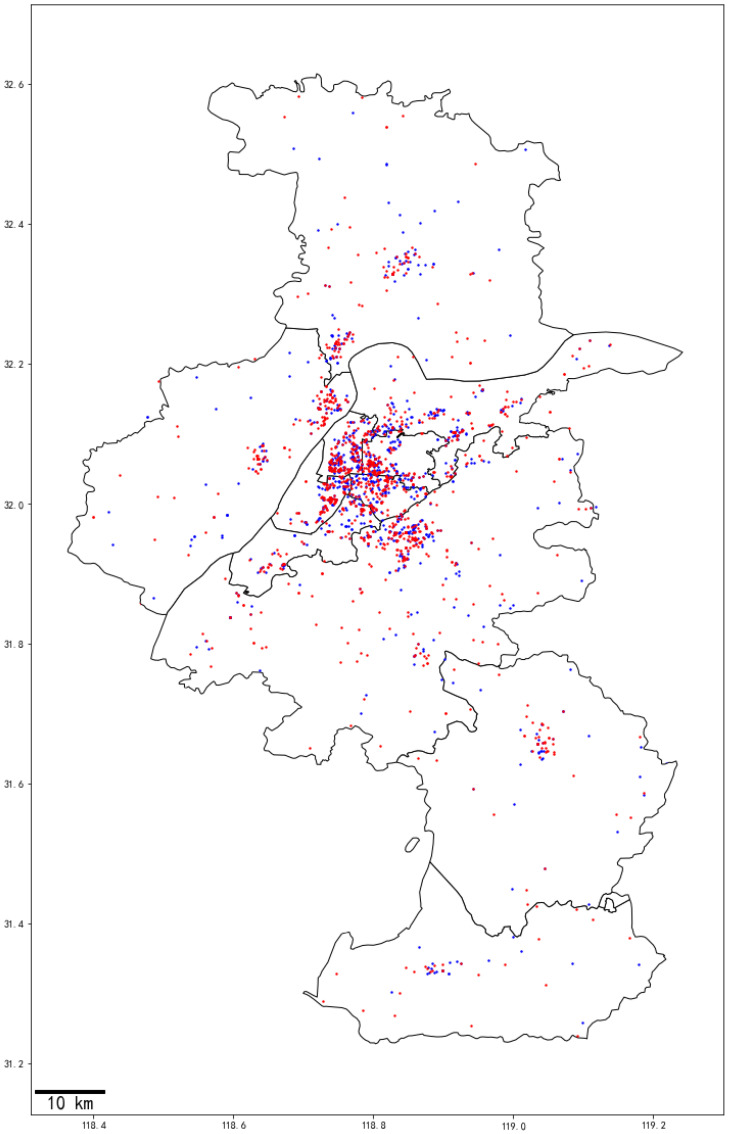
Distribution of the geographical locations of the participants in Nanjing, China. The blue dots are simple breast/testes development and the red dots are central precocious puberty.

The Institutional Review Board of the Children’s Hospital of Nanjing Medical University approved this study (202108080-1). All studies have been performed in accordance with relevant guidelines and regulations. From January 2016 to December 2020, 2201 children diagnosed with precocious puberty by a professional pediatrician as well as permanent residents of Nanjing were included in the study. Data used in this study were anonymous, and no individually identifiable information was available. Data on gender and age were obtained from hospital records. The distribution of the geographical locations of the cases is shown in Figure 1.

### 2.2. Air Pollution Exposure

Nanjing, located in Jiangsu Province and the Yangtze River Delta, not only has a developed industrial economy but also represents a typical city of China. It also reflects the degree of general urban air pollution in the eastern China area well. There are nine national air quality monitoring locations in Nanjing, including Xianlin University Town, Caochangmen, Xuanwu Lake, Zhonghuamen, Ruijin Road, Shanxi Road, Maigaoqiao, Pukou, and Olympic Sports Center, which represent the cultural area, ecological park district, traffic area, residential area, business area, industrial zone, suburb area, and new districts, respectively.

We obtained the daily 24 h monitor data of PM_2.5_, PM_10_, SO_2_, NO_2_, CO, and O_3_ from all of the national air quality monitoring stations in Nanjing from August 2015 to December 2021. According to the home address of each case diagnosed from January 2016 to December 2020, exposure levels of air pollution in 140 days of each case before diagnosis were calculated. The exposure level of six different types of air pollution at each participant’s residence was predicted by using the inverse distance weighting (IDW) method, which is a commonly used spatial interpolation method to model distribution from data points and is widely used for air pollution research [15].

### 2.3. Measurements of Children’s Precocious Puberty

Precocious puberty is defined as the onset of secondary sexual characteristics that appear in girls before 8 years old, or in boys before 9 years old. The precocious puberty in the study is generally divided into two types based on whether the hypothalamic–pituitary–gonadal (HPG) axis function is activated in advance, simple breast/testes development, and central precocious puberty. The diagnostic criteria for simple breast/testes development include: (1) Secondary sexual characteristics appear in girls before 8 years old, or in boys before 9 years old. (2) The development of sexual characteristics does not progress according to the normal development procedure. (3) Gonadotropin levels are both at the pre-pubertal level. The diagnostic criteria of central precocious puberty include: (1) Secondary sexual characteristics appear in girls before 8 years old, or in boys before 9 years old. Breast nodules in girls and increased testicular volume in boys were considered as the first manifestations. (2) The annual growth rate with linear acceleration is higher than that of normal children. (3) Bone age exceeds actual age by 1 year or more. (4) B ultrasound showed that the volume of the uterus and ovary increased, and multiple ovarian follicles with a diameter of more than 4 mm were visible in the ovary. The testicular volume of the boy was more than 4 mL. (5) HPGA function started, and in an LHRH stimulation experiment, the LH/FSH peak value was greater than 0.6, and the LH peak value was greater than or equal to 5.0 U/L. We excluded secondary central precocities, such as central nervous system occupying, infection, trauma, postoperative, radiotherapy or chemotherapy, and congenital dysplasia. In addition, other primary diseases which may lead to precocious puberty were excluded as well, such as congenital adrenal hyperplasia, McCune–Albright syndrome, and precocious puberty associated with congenital hypothyroidism.

### 2.4. Statistical Analysis

We analyzed precocious puberty based on a case-crossover design. This method matches exposure at or shortly before the event with multiple periods [16,17,18]. We took the 4 weeks (0–28 days) before the onset of precocious puberty as case days and matched 4 control days for each case day, which were the same day at 5–8 weeks, 9–12 weeks, 13–16 weeks, and 17–20 weeks before the onset, respectively. For example, if precocious puberty occurred on a Monday of the year, the control group included 4 weeks ago, 8 weeks ago, 12 weeks ago, and 16 weeks ago on 4 different Mondays.

Previous studies have shown that the relationship between air pollution exposure and health outcomes is non-linear, and there is a significant lag effect in this relationship. Gasparrini built a statistical framework of a Distributed lag nonlinear model (DLNM) based on cross-basis functions and modeled nonlinear expose-response and hysteresis relations [19,20]. In this study, we adopted a DLNM with logistic regression to calculate the odds ratio (OR) and 95% confidence interval (CI) of the lag effect of PM_2.5_ and PM_10_ on precocious puberty with the threshold of fine particle pollution of 150 μg/m^3^ as a reference point (threshold value defining the heavily polluted days according to the National Ambient Air Quality Standard in China, GB 3096-2012). The maximum lag day in our analysis was set to 28 days to estimate the overall cumulative effects. The model was adjusted for the age, SO_2_, NO_2_, CO, and O_3_ levels on the same day. In a subgroup analysis, the participants were classified into two types, simple breast/testes development, and central precocious puberty. Descriptive statistics were carried out in order to give the characteristic of the data and all statistical tests were two-sided. The “dlnm” package was used to create the DLNM model. All analyses were performed by using R software (version 4.1.3, R Development Core Team, Vienna, Austria).

## 3. Results

From January 2016 to December 2021, a total of 2201 children who were diagnosed with precocious puberty have been included in the dataset, including 74 boys and 2127 girls (Table 1). The incidence rate of girls was much higher than that of boys. The average age of children was 7.47 ± 1.24 years old. By analyzing the onset time of children diagnosed with precocious puberty, the incidence of precocious puberty is on the rise. Among these children, there were 1178 children diagnosed with central precocious puberty and 1023 children diagnosed with simple breast/testes development. Children who were diagnosed with simple breast/testes development were slightly younger than those who were diagnosed with central precocious puberty (*p* < 0.001). Characteristics of air pollution are shown in Table 2. The average concentration and standard deviation of each air pollution variable was 38.81 ± 26.36 μg/m^3^ for PM_2.5_, 69.77 ± 41.07 μg/m^3^ for PM_10_, 11.47 ± 6.76 μg/m^3^ for SO_2_, 39.94 ± 17.27 μg/m^3^ for NO_2_, 0.86 ± 0.31 mg/m^3^ for CO, and 71.26 ± 31.43 μg/m^3^ for O_3_. The max concentration of PM_2.5_ and PM_10_ were 285.75 μg/m^3^ and 447.39 μg/m^3^, respectively.

**Figure 2:**
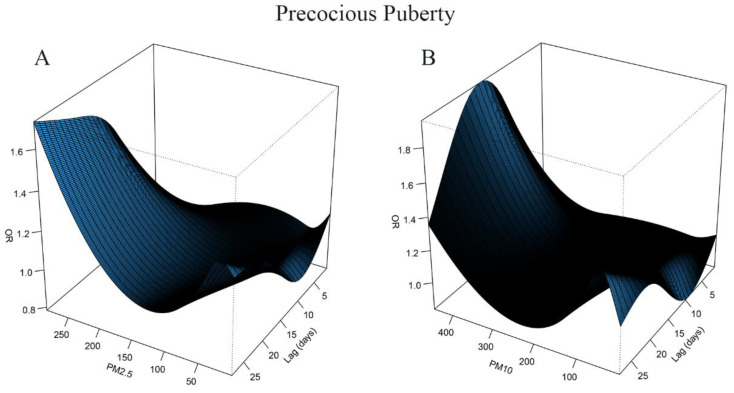
The three-dimensional (3D) plots of lag associations up to 28 days of PM_2.5_ (**A**) and PM_10_ (**B**) exposure with odds ratios (OR) of precocious puberty.

**Figure 3:**
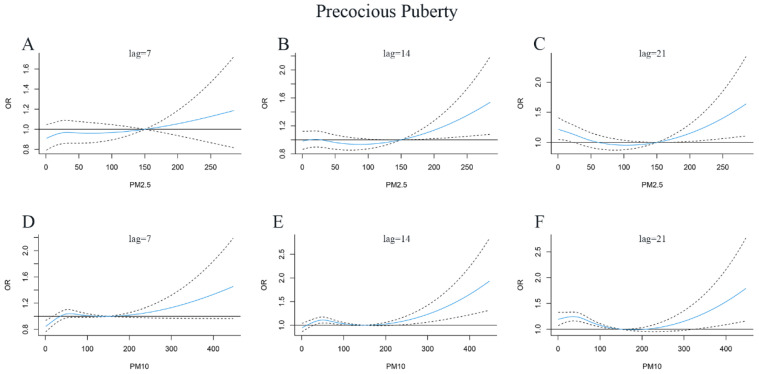
Lag-specific odds ratio (OR) and 95% confidence interval (CI) of precocious puberty with up to 28 days of exposure to PM_2.5_ and PM_10_. (**A**): PM_2.5_, lag = 7, (**B**): PM_2.5_, lag = 14, (**C**): PM_2.5_, lag = 21, (**D**): PM_10_, lag = 7, (**E**): PM_10_, lag = 14, (**F**): PM_10_, lag = 21. The dotted line and solid line indicate OR and 95% CI, respectively.

We found a nonlinear dose-response relationship between PM_2.5_, PM_10_ exposure, and the risk of precocious puberty using the DLNM model adjusted for age, gender, SO_2_, NO_2_, CO, and O_3_ levels. We used three-dimensional (3D) plots to visualize the exposure–lag–response relationship of PM_2.5_, PM_10_ exposure, and the risk of precocious puberty (Figure 2). For the cross basis of PM_2.5_, PM_10_ exposure, and other air pollution, we used natural cubic splines for the exposure and lag space. The parameter setting chosen was three degrees of freedom for both lag and exposure splines. The results showed that the higher the PM_2.5_ concentration, the longer the lag time, and the greater the risk of precocious puberty. When the samples were exposed to high concentrations of PM_10_, the risk of precocious puberty was greatest with a lag of about 16 days. The strongest effects of the PM_2.5_ and PM_10_ on precocious puberty were observed in lag27 (OR = 1.72, 95% CI: 1.01–2.92) and lag16 (OR = 1.95, 95% CI: 1.33–2.85), respectively. The associations between PM_2.5_, PM_10_ exposure, and the risk of precocious puberty at specific lag days are shown in Figure 3.

**Figure 4:**
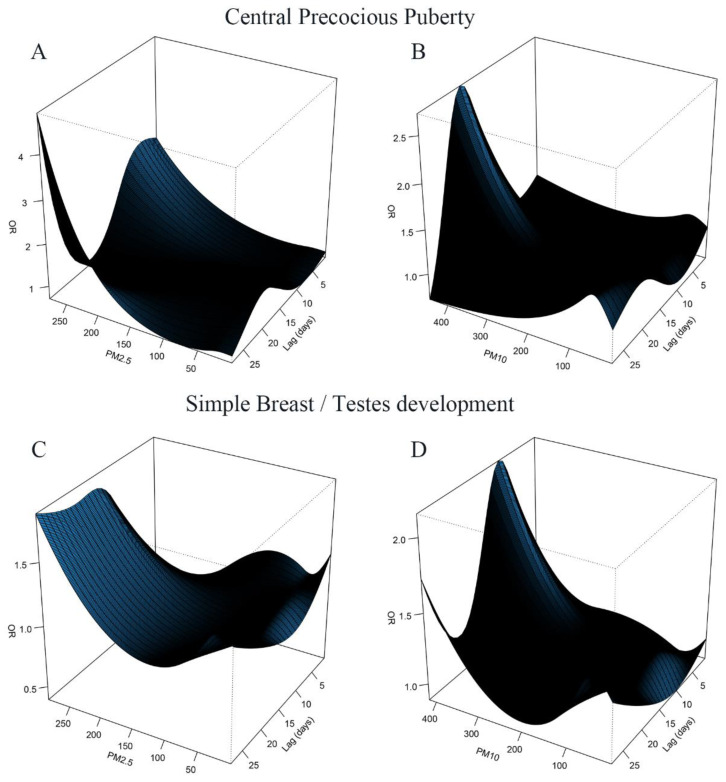
The three-dimensional (3D) plots of lag associations up to 28 days of PM_2.5_ (**A**,**C**) and PM_10_ (**B**,**D**) exposure with odds ratios (OR) of central precocious puberty and simple breast/testes development.

**Figure 5:**
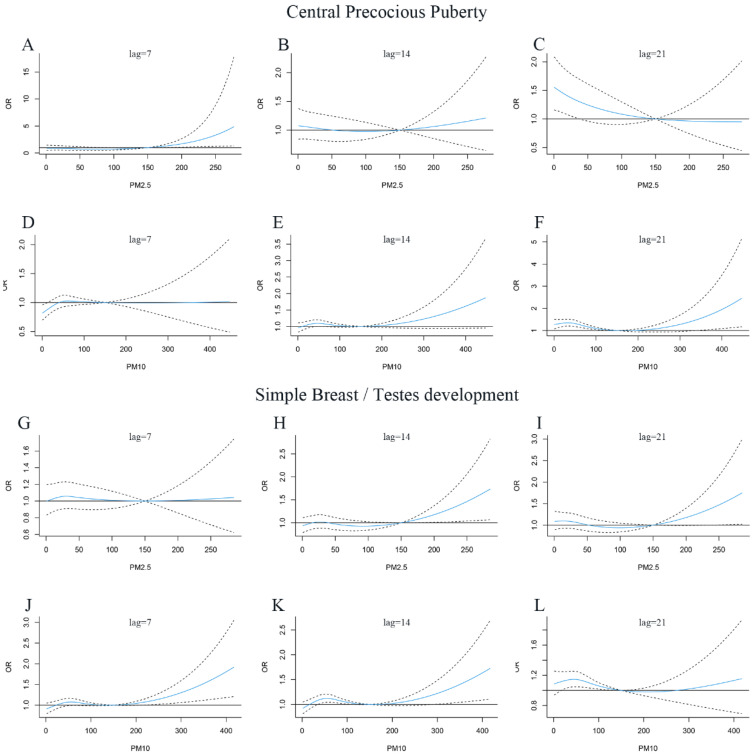
Lag-specific odds ratio (OR) and 95% confidence interval (CI) of central precocious puberty and simple breast/testes development with up to 28 days of exposure to PM_2.5_ and PM_10_. (**A**): Central precocious puberty, PM_2.5_, lag = 7, (**B**): Central precocious puberty, PM_2.5_, lag = 14, (**C**): Central precocious puberty, PM_2.5_, lag = 21, (**D**): Central precocious puberty, PM_10_, lag = 7, (**E**): Central precocious puberty, PM_10_, lag = 14, (**F**): Central precocious puberty, PM_10_, lag = 21. (**G**): Simple breast/testes development, PM_2.5_, lag = 7, (**H**): Simple breast/testes development, PM_2.5_, lag = 14, (**I**): Simple breast/testes development, PM_2.5_, lag = 21, (**J**): Simple breast/testes development, PM_10_, lag = 7, (**K**): Simple breast/testes development, PM_10_, lag = 14, (**L**): Simple breast/testes development, PM_10_, lag = 21. The dotted line and solid line indicate OR and 95% CI, respectively.

As a stratification analysis, the relationship between air pollution and the risk of central precocious puberty and simple breast/testes development were established, respectively. We observed a similar effect for both categories (Figure 4 and Figure 5). It was found that when the lag time was more than 20 days, the high concentration of PM_2.5_ exposure had a great impact on the risk of central precocious puberty. The lag time of high concentration of PM_10_ on the risk of simple breast/testes development was 10 days, while the lag time of PM_10_ on the risk of central precocious puberty was extended to 20 days.

## 4. Discussion

In China, the incidence of precocious puberty in children has been rising in recent years, while negative impacts on children’s physical and mental health cannot be ignored [21,22]. The mean age of the children who were diagnosed with precocious puberty was 7.47 ± 1.24 years old, which is consistent with most previous studies [21,23,24]. Among this representative population in Nanjing, our analyses suggested that ambient air pollution was significantly associated with the risk of precocious puberty. Lagged effects were observed for heavily polluted days. Depending on the type of precocious puberty, we have done a stratification analysis. The correlation model between air pollution and the risk of central precocious puberty and simple breast/testes development have been successfully established. The results showed that there were few differences between the two kinds of influence mechanisms of atmospheric particulate pollution on central precocious puberty and simple breast/testes development. At the public health level, the impact of our findings might be illustrated by previous studies showing that precocious puberty is associated with health problems, such as psychological behavior changes and fusion of epiphyseal resulting in short adult stature [24,25,26]. Our study adds to the literature on the lag associations between ambient air pollution and the risk of precocious puberty. The finding provides more epidemiological evidence for future mechanism studies on how ambient air pollution affects human health.

We assessed ambient exposure levels of PM_2.5_ and other air pollution before precocious puberty was diagnosed. The mean PM_2.5_ exposure in our study was 38.81 ± 26.36 μg/m^3^, which is approximately three-fold higher than the WHO air quality guideline (the exposure level is recommended to not exceed an annual mean of 10 μg/m^3^). After comparing the mean PM_2.5_ exposure in our study with that in other studies, we found that it was at the same level as or lower than that in developing countries [27], and higher than that in developed countries [28]. Due to consideration of the time, cost, and technical factors, measuring the concentration of air pollutants for each individual using personal wearable devices is infeasible among large populations [29]. Assessing links between air pollution and health is complicated by the difficulty of properly measuring air pollution exposures to the human body [30]. The exposure monitor is a commonly used method of assessing ambient air pollution exposures, but it is limited by the number and placement. As moving individuals were exposed to a variety of air pollution every day, the estimated ambient air pollution using the IDW method based on the home address of each case and air quality monitoring locations in our study may not be accurate enough to reflect the human body’s perception of exposure to the external environment. Most of those children diagnosed with precocious puberty are school-age. The inclusion of individual assessments of ambient air pollution exposure in the school would lead to more accurate results. In addition, the patterns of indoor exposure to air pollutants might play an important role in the development of children’s diseases [31,32]. As such, the development of new tools to assess air pollution exposures that meet the accuracy assessment of individual exposure would also be needed.

A predictable finding in the study was that there were several days of lag for PM_2.5_ and PM_10_ exposure to take effect. The demonstrated lag that other studies and we have found between PM_2.5_ exposure and the onset of symptoms could be indicative of the period of time necessary for PM_2.5_ exposure to result in a buildup of toxicity and for a chronic immune response to occur [33,34]. Furthermore, these manifestations are occurring against the background of the gradual maturation process of both the immune and reproductive systems of children. According to our study, we may conclude that exposure to high concentrations of air pollution has a significant effect on the risk of precocious puberty. To the best of our knowledge, only two previous studies have analyzed the effect of air pollution and child sexual development in the literature, reporting similar results. Both studies concluded that exposure to air pollution could influence the timing of pubertal development. In one of the studies, a total of 639 girls were analyzed in search of a possible association between pre-menarcheal exposure to PM_10_ and age at menarche in South Korea by using data from the fifth Korea National Health and Nutrition Examination Survey (KNHANES V) [35]. The authors reported that exposure to elevated PM_10_ concentration has a significant effect on decreasing age at menarche. In another newer study, Polish researchers have incorporated more air pollutants, i.e., particulate matter (PM_10_, PM_2.5_), sulphur dioxide, nitric oxide, and benzene [36]. It was found that the air pollutants PM_10_, PM_2.5_, and NO negatively affect the age at menarche, also after adjusting for socioeconomic status.

Numerous studies have proved that air pollution has detrimental effects on many diseases such as cardiovascular and respiratory disease, diabetes, cancer, autoimmunity, and epilepsy [37,38]. Our results show that air pollutants were associated with the risk of precocious puberty. The related action mechanism is still not explored specifically in the human population. Evidence has shown that PM_2.5_ contains a strong oxidant and produces reactive oxygen species, and we hypothesized that the effects of oxidative stress may increase the risk of precocious puberty [39,40]. The polycyclic aromatic hydrocarbons and heavy metals contained in particulate matter, especially from fossil fuel combustion, are described in some literature as endocrine disruptors through activation of the aryl hydrocarbon receptor, androgen, or estrogen receptors [41,42,43,44]. In addition, exposure to PM_2.5_ might stimulate the endocrine system and induce increased circulation of stress hormones, leading to damage to fat, muscle tissue, and some organs [45,46,47]. These factors may lead to precocious puberty in a variety of ways. The effects of air pollution on the sexual development system need to be further explored and verified.

Our research has several strengths. To the best of our knowledge, this is the first study that air pollutants were independently associated with the risk of precocious puberty using a distributed, non-linear model. The distribution of the delayed effect can be specified at different time points. Second, we further explored the effects of PM_2.5_ and PM_10_ on the risk of central precocious puberty and simple breast/testes development. The results showed there were few differences between the two kinds of air pollution on sexual precocity. Third, the case-crossover design was used in our study. It could provide a reliable effect to estimate through matching cases and effectively controls the confounding of individual-level characteristics [18,48]. Moreover, considering the potential confounding effects of other air pollution on sexual precocity, we adjusted our model for co-air pollutants SO_2_, NO_2_, CO, and O_3_ in the model to reduce the confounding effects.

Despite the novelties and strengths of the present study, several limitations must be acknowledged. This is a single-center study and the result may not be well generalizable and may lead to potential selection bias. However, our hospital is the only university-affiliated children’s hospital in Nanjing, and most children with precocious puberty were treated in our hospital. It was a good sampling of the overall population with precocious puberty in Nanjing city. Second, we estimated PM_2.5_ exposure only based on the home address from hospital records by using the IDW method. The calculated air pollution exposure levels were not accurate enough exposure levels for an individual patient. Thirdly, although our study controlled some potential confounding factors, we did not seek the family history of parental precocity and other meteorological factors, such as ultraviolet intensity, sunshine duration, and ambient temperature, which may also play a role in the development of precocious puberty. Thus, whether acute or long-term exposure to PM_2.5_ and PM_10_ or their components affect the risk of precocious puberty remains more evidence to be determined.

## 5. Conclusions

Ambient air pollution exposure might play a role in precocious puberty. A similar effect was observed both in central precocious puberty and simple breast/testes development. Air pollution is a manageable and improvable environmental risk factor, and our results provide a theoretical basis for policy aimed at protecting children from air pollution.

## Figures and Tables

**Table 1:** Characteristics of 2201 children living in Nanjing with precocious puberty.

	Precocious Puberty	Central Precocious Puberty	Simple Breast/Testes Development
N	2201	1178	1023
Age	7.47 ± 1.243	7.71 ± 1.036	7.19 ± 1.394
Gender			
Boys (%)	74 (3.36)	45 (3.82)	29 (2.83)
Girls (%)	2127 (96.64)	1133 (96.18)	994 (97.17)

**Table 2:** The distribution of exposure to ambient air pollution.

Variable	Mean ± SD	Min	5%	25%	50%	75%	95%	Max
PM_2.5_ (μg/m^3^)	38.81 ± 26.36	0.04	11.42	21.52	32.24	47.88	89.91	285.75
PM_10_ (μg/m^3^)	69.77 ± 41.07	0.72	23.39	41.72	60.25	87.88	146.03	447.39
SO_2_ (μg/m^3^)	11.47 ± 6.76	1.05	4.10	6.62	9.76	14.46	24.82	110.04
NO_2_ (μg/m^3^)	39.94 ± 17.27	3.06	18.46	27.43	36.37	48.97	73.61	153.2
CO (mg/m^3^)	0.86 ± 0.31	0.02	0.45	0.66	0.81	1.01	1.43	7.30
O_3_ (μg/m^3^)	71.26 ± 31.43	3.05	23.55	48.72	69.46	90.56	127.97	250.68

## Data Availability

The datasets used and/or analyzed during the current study are available from the corresponding author upon reasonable request.
